# Tracking low back pain in adolescent idiopathic scoliosis: a prospective cohort study protocol

**DOI:** 10.1186/s12998-017-0155-x

**Published:** 2017-09-05

**Authors:** Jean Théroux, Norman Stomski, Christopher J. Hodgetts, Charlotte Leboeuf-Yde, Bruce F. Walker, Sylvie Le May, Hubert Labelle

**Affiliations:** 1 Research Center, Sainte-Justine University Hospital Center, Montreal, QC Canada; 2 School of Health Professions, Murdoch University, 90, South Street, Murdoch, WA 6150 Australia; 3 Faculty of Nursing, University of Montreal, Montreal, QC Canada; 4 Faculty of Medicine, University of Montreal, Montreal, Canada

**Keywords:** Low back pain, Adolescent idiopathic scoliosis, Disease burden, Prospective, Prevalence

## Abstract

**Background:**

Numerous methodological limitations have constrained the findings of previous studies that have examined the prevalence of low back pain in adolescents with idiopathic scoliosis. This article presents a study protocol that has been designed to address the shortcomings of prior research in this area. In addition, it will establish the level of disease burden associated with acute, recurrent, and chronic low back pain in adolescents with idiopathic scoliosis.

**Methods:**

This study will involve a prospective cohort of adolescents with idiopathic scoliosis presenting to an outpatient department in a paediatric hospital. Potential participants will be eligible for inclusion if they are aged 10–17 years, experience adolescent idiopathic scoliosis, own a mobile phone, and are able to communicate in either French or English adequately. The primary outcome measure is the presence of low back pain. The secondary outcome will be measures with the Brief Pain Questionnaire and the PedsQL questionnaire. Participants will be followed over a 12-month period reporting weekly, via SMS-tracking.

**Discussion:**

Previous studies frequently established the prevalence of low back pain through asking participants to recall whether they experienced low back pain over certain periods. These periods often extended beyond many months, and hence were subject to recall bias. Our study addresses such bias through gathering data on a weekly basis using SMS-tracking providing detailed information about the progression of low back pain, which allows researchers to establish the prevalence of acute, recurrent, and chronic low back pain with a better certainty. Furthermore, the previous studies failed to use a standardised definition of low back pain. As such, it is not possible to determine whether the reported low back pain was experienced at the following standardised defined location: “pain in the space between the lower posterior margin of the rib cage and the horizontal gluteal fold”.

**Conclusion:**

This research protocol will be the first study to determine the proportion of adolescents with idiopathic scoliosis who experience acute, recurrent, and chronic low back pain, and establish the level of the burden associated with these subgroups of low back pain.

## Background

Adolescent Idiopathic Scoliosis (AIS) is defined by the Scoliosis Research Society (SRS) has a three-dimensional deviation of the vertebral column of unknown origin [1] appearing in adolescents older than 10 years. This deviation is associated with a Cobb angle (measurement in degrees of the scoliosis deviation on a posterior-anterior radiograph) greater than 10 degrees, and its prevalence is estimated to vary from 2 to 3% [2, 3]. Adolescent idiopathic scoliosis has generally been perceived as a non-painful condition [4, 5] and research has mainly focussed on aetiological factors [6], conservative treatment such as bracing [7], and surgical procedures [3]. Only recently have researchers looked at back pain in adolescents with idiopathic scoliosis and found that it was more prevalent than in healthy adolescents [8].

To better understand this issue, we recently conducted a systematic review [9] of prevalence studies of low back pain in adolescents with idiopathic scoliosis. We found that only two studies [10, 11] had been undertaken in this area and that several methodological limitations constrained the findings of these studies. The primary limitations included: 1) use of non-standardised anatomical definitions of low back pain; 2) lack of low back pain episode definitions; 3) and lack of longitudinal monitoring preventing the establishment of whether cases of low back pain were acute, brief and transitory, or if these cases were recurrent or chronic.

### Importance of understanding the progression of low back pain for adolescents with idiopathic scoliosis

As previously noted, no previous studies have established the prevalence of acute, recurrent, and chronic low back in adolescents with idiopathic scoliosis. As such, it is unknown if the level of disease burden varies across adolescents with idiopathic scoliosis who experience different types of low back pain. Therefore, it is important to understand if acute, recurrent, and chronic low back pain substantially, as well as the absence of low back pain, differ in terms of burden in adolescents with idiopathic scoliosis. This knowledge could be used to deliver tailored interventions to specific individuals who are most likely to experience significant disability.

### Impact of low back pain in the general adolescent population

In the general adolescent population, chronic low back pain results in significantly higher levels of disease burden in comparison to non-chronic low back pain [12]. Such burden includes increased use of professional health care, higher levels of medication use, more missed school and work days, increased psychosocial distress, and higher levels of interference with normal and physical activities [12]. Furthermore, adolescents with chronic low back pain are four times more likely to experience low back pain in adulthood when compared to adolescents with non-chronic low back pain [13, 14].

### Potential aetiological low back pain factors

Low back pain, whether in adolescents or adults, can be caused by different structures as long as these structures are innervated, able to cause pain, and are prone to diseases or injuries known to be painful [15]. Commonly implicated structures are the intervertebral disc, the facet joint and the sacroiliac joint. [16–18]. In adolescent idiopathic scoliosis patients, low back pain appears to be more prevalent compared to healthy adolescents [8]. Considering the structural effects that spinal deformities, such as scoliosis, may have on the intervertebral discs, the facet joints, or the sacroiliac joint, it is conceivable that these structures would be at a greater risk of injury [19, 20]. Unfortunately, these structures have mainly been researched regarding their relationship with the development and progression of scoliosis [21–24]. Thus, the higher reported low back pain prevalence in adolescents with idiopathic scoliosis compared to non-scoliosis adolescents [8].

### Defining low back pain

Defining low back pain is relatively straightforward, yet many researchers neglect essential details which confound the reporting of low back pain prevalence studies. The first step in reporting low back pain involves the presentation of a standardised definition for the anatomical location of low back pain. A consensus developed guidelines uniformly state that low back pain involves the occurrence of pain in the space between the lower posterior margin of the rib cage and the horizontal gluteal fold [25]. This location is often best explained to research participants through illustrated diagrams.

After stating the anatomical location, it is then necessary to define what constitutes an episode of low back pain so that these episodes can be further classified into acute, recurrent, and chronic episodes. De Vet’s [26] definition of an episode of low back pain is the most explicit available definition and it is also the definition we will use in our proposed project. This definition states that an episode of low back pain is a period of low back pain lasting more than 24 h preceded and separated by a period of at least 1 month without low back pain. It should also be noted that to qualify for an episode of low back pain, the minimum pain intensity experienced over 24 h must correspond to the minimum clinically important change for a particular scale [26].

In light of the above definition, low back pain can then be segmented into acute, recurrent, or chronic episodes. Acute low back pain involves an episode that does not persist for more than 3 months, whereas chronic low back pain comprises an episode that lasts for more than 3 months. Recurrent low back pain consists of at least two discrete episodes that occur within a 12 month period.

As we have shown above, it is important to define the anatomical location of low back pain. It is also important to correctly classified episodes of low back pain. Unfortunately, we found that none of the studies that examined low back pain in adolescents with scoliosis had provided these details [4, 8, 10, 27, 28].

Our proposed program of research will address the methodological limitations in previous studies by using standardised low back pain case definitions in monitoring low back pain in adolescents with idiopathic scoliosis at weekly intervals over a 12-month period. As such, this proposed research program will aim to establish the prevalence of acute, recurrent, and chronic low back pain in adolescents with idiopathic scoliosis. Moreover, our proposed research will also seek to determine whether acute, recurrent, and chronic low back pain substantially differ in terms of the burden in adolescents with idiopathic scoliosis. Such information could significantly inform the delivery of health services for adolescents with idiopathic scoliosis.

### Central hypothesis

Adolescents with idiopathic scoliosis will frequently experience chronic low back pain, and chronic low back pain will be associated with substantially higher levels of disease burden when compared to the burden that results from acute or recurrent low back pain.

### Research aims

The overall objective of this proposed prospective cohort study is to report the pattern of low back pain (acute, recurrent, or chronic) over a 12-month period in adolescents with idiopathic scoliosis. More specifically, this study aims to 1) identify the prevalence of acute, recurrent, and chronic low back pain in adolescents with idiopathic scoliosis, and to 2) identify the level of disease burden associated with acute, recurrent, and chronic low back pain in adolescents with idiopathic scoliosis.

## Methods

### Study design

A prospective cohort study of adolescents with idiopathic scoliosis presenting to an outpatient department in a paediatric hospital. This study protocol complies with the recommendations for observational studies set out in the STROBE guidelines [29].

### Sample and setting

The Sainte-Justine Hospital is one of the largest paediatric hospitals in Canada and provides care to almost half of the Quebec paediatric population. It receives adolescents for evaluation from major cities of the Québec provinces [30]. Adolescents with an observed spinal abnormality noted by a parent or other health professionals are referred to CHU Sainte-Justine to receive a confirmatory diagnosis. A recent study [30] found that 3900 patients visited the Sainte-Justine spinal deformity clinic over a 1-year period, of whom almost one-third received a diagnosis of idiopathic scoliosis. We intend to recruit consecutively approximately 750 patients with a confirmed diagnosis of scoliosis from the spinal deformity clinic of the Sainte-Justine hospital over a 12-month period. Considering the volume of patients seen at this clinic yearly, we are confident that we will be able to recruit the required sample size.

### Ethics

Ethical approval will be obtained from both the Sainte-Justine Hospital and the Murdoch University Human Research Ethics Committee.

### Recruitment process

Eligible participants will be consecutively recruited through only the Sainte-Justine hospital. All eligible participants will be invited to participate by a research nurse upon attendance at the Sainte-Justine Hospital. No other sources will be used to recruit participants. Written informed consent will be obtained from potential participants and parent/legal guardian before the potential participants are enrolled in the study.

### Inclusion criteria

Potential participants attending the Sainte-Justine University teaching hospital orthopaedic department will be eligible to participate if they have been diagnosed with adolescent idiopathic scoliosis as defined by the Scoliosis Research Society [31]. This diagnosis will be documented in the patient record by their attending orthopaedic physician. In addition, to be eligible for inclusion potential participants must be aged 10–17 years, own a mobile phone, and able to communicate competently in either French or English.

### Exclusion criteria

Potential participants will be excluded from this study if they have spinal deformities other than adolescent idiopathic scoliosis, spinal pathologies, experience mental disabilities, undergone back surgery, or suffered significant physical trauma in the prior 12 months.

### Sample size

This study will establish the proportion of adolescents with idiopathic scoliosis who experience acute, recurrent, and chronic low back pain. The prevalence of general back pain in adolescents with idiopathic scoliosis has been estimated at 50%. If acute, recurrent, and chronic low back pain are evenly distributed among those who experience back pain, then a sample of 750 participants provides 95% confidence interval width of 0.09 (considering a two-sided confidence interval for one proportion confidence interval formula: simple asymptotic with continuity sample).

### Instruments

#### Primary outcome

The primary outcome in this study, the presence of low back pain, will be assessed with the following question: “In the last week have you experienced low back pain more or less continuously for about a day (yes/no)”. This question accords with the de Vet’s criterion for the initiation of a low back pain episode, but it has not been validated in an adolescent, or indeed adult, population. Hence, we will undertake a content validity study to assess the relevance and comprehensibility of this question, which will be conducted as follows. The original English question will be translated into French using a reverse double translation method. Five English, and five French, experts (adolescents) will be recruited to respectively assess the content validity of the English and French versions of the questionnaire item. The item content validity or I-CVI (proportion of participants who agree with a score of 3 or 4) and the Kappa will be calculated [32, 33]. The item will be modified if the obtained value for the CVI and the Kappa are respectively less than 0.80 and 0.75.

#### Secondary outcomes

Secondary outcomes in this study will be assessed with the Brief Pain Inventory and PedsQL. These instruments are described in detail in the following section.

The Brief Pain Inventory (BPI) is used to evaluate the quality and the intensity of pain and determine its impact upon the patient [34]. The BPI includes 9 questions and uses an 11 -point scale (0 = “no pain”; and 10 = “pain as bad as you can imagine”) to evaluate the intensity of pain at the time of being surveyed, pain at its worst, pain at its least, and average pain in the past week. Scores are calculated by averaging items that comprise each scale. A higher score indicates a greater pain-related interference. This instrument also records the location of pain on a diagram of a human silhouette and requires patients to select words that best describe their pain. This questionnaire takes about 10 min to fill out. It has demonstrated validity and reliability in multiple cultures and languages [35–38]. Even if this instrument was initially developed to evaluate pain in cancer patients, it has been validated in other conditions [39, 40] as well as low back pain [41].

The PedsQL assesses health-related quality of life, over the last month, across the following four domains: physical, emotional, social, and school functioning [42]. Items are reverse-scored and linearly transformed to a 0 to 100 scale (0 = 100, 1 = 75, 2 = 50, 3 = 25, 4 = 0). A high score indicates a better quality of life. Further, a physical score, a psychological score and a global score are also obtained. The psychological score is derived from the average score of the emotional, social and school functioning domains; the physical score corresponds to the average score of the physical domain; finally, the total score corresponds to the average of each domain. The PedsQL has been validated in French-Canadian [42, 43] adolescent populations and demonstrates adequate levels of reliability. This instrument requires only 5 min to complete. It has been shown to distinguish between acute and chronic conditions and is especially useful in determining disease severity.

### Data collection

The Sainte-Justine Hospital spinal deformity clinic is a major paediatric research centre. Patients are accustomed to completing outcome measures each time they visit the deformity clinic. At the initial appointment, after receiving a confirmatory diagnosis of adolescent scoliosis, participants will complete the BPI questionnaire and the PedsQL and the following demographic details will also be captured: age, gender, menarchal status, use of a brace, Cobb angle, and angle of trunk inclination. Participants will then be enrolled onto the SMS tracking system to be able to receive weekly text messages. Thereafter, the presence of low back pain data will be gathered at weekly intervals via SMS (phone text messages) sent to participants, which has been shown to result in high response rates in adolescent populations [44]. Each SMS message will contain one question: “In the last week have you experienced low back pain more or less continuously for about a day (yes/no)”. The SMS messages will be distributed by SMS-track [45], which is a company that has been widely used by epidemiologists. A first automatic reminder will be sent if the SMS reply has not been received 2 days after the initial SMS and if necessary a second reminder will be sent 1 day after the first reminder. If no answer has been received after that period of time, participants will be phoned to remind them to answer the SMS. The SMS data is automatically uploaded to a data file that the research team can access. In addition, at 12-month follow-up, the research team will send a link that will enable all participants to complete an online version of PedsQL questionnaire. Data from the PedsQL questionnaire will provide information about the participants’ level of disease burden.

### Statistical analysis plan

Data will be entered and analysed in SPSS v.22. Where appropriate, missing data will be handled with multiple imputations. Since it is uncertain whether missing data will be monotonic, discriminant function imputation will be used in preference to logistic regression imputation. The data captured through weekly SMS messages will be examined descriptively to identify the proportion of adolescents with idiopathic scoliosis who experience acute, recurrent, and chronic low back pain, using the following definitions:

- episode of low back pain: a period of low back pain on at least 1 day in a given week preceded and separated by a period of at least 1 month without low back pain.
- acute low back pain: an episode that does not persist for more than 3 months; chronic low back pain: an episode that lasts for more than 3 months.
- recurrent low back pain: at least two discrete episodes that occur within a 12-month period.

Finally, the level of disease burden, captured by administration of PedsQL, will be reported descriptively for the acute, recurrent, and chronic low back pain subgroups. ANCOVA will be used to examine differences in disease burden between the low back pain subgroups. Demographic and clinical characteristic data collected at baseline will be entered as confounder/moderator variables in the ANCOVA model. The selection of confounder/moderator variables will be based on a descriptive comparison between low back pain sub-groups (no pain, acute, recurrent, chronic) of demographic and clinical characteristic values to identify important differences that may result in confounding effects.

## Discussion

We elected to gather information about the prevalence of low back pain by using SMS-Track administered at weekly intervals. Such data provides highly detailed information about the progression of low back pain, which allows researchers to establish the prevalence of acute, recurrent, and chronic low back pain with a high degree of certainty. Previous studies frequently established prevalence through asking participants to recall whether they experienced low back pain over certain periods [46]. These periods often extended beyond many months, and hence were subject to recall bias [47]. Our study addresses such bias through gathering data on a weekly basis.

In prospective studies, instruments are commonly administered only at baseline and follow-up [48]. Change in the instruments between these time points is thought to capture change in the condition under assessment. But administering instruments at several discrete time points may not capture change in conditions that vary from day to day [49]. On the one hand, a difference between instruments may reflect momentary variation in a stable condition. Alternatively, lack of change in instruments would indicate no change in the condition, but the condition might have been better or worse in the period between outcome assessments. This has implications for the studies of low back pain, as it has been recognised as a condition that typically fluctuates in its course [50, 51]. The design of our study addresses this issue through obtaining details about low back pain once per week.

Prior low back pain studies in adolescents with idiopathic scoliosis failed to specify the anatomic location of low back pain [9]. As such, it is not possible to determine whether the reported low back pain was experienced at the following standardised defined location: “pain in the space between the lower posterior margin of the rib cage and the horizontal gluteal fold”. Most adolescents would probably not understand the location of pain when expressed in those terms. It is, therefore, important to provide adolescents with a visual aid such as a mannikin with a shaded area that clearly demarcates the location of low back pain. Subsequently, we will ensure that at the baseline assessment all participants and parents/guardians are provided with visual aids to take home.

One of the common limitations of prospective cohort studies is drop out rates, particularly when studies are conducted over a 12 month period [52]. Losses to follow-up may affect statistical power but are otherwise inconsequential if they are random [48]**.** But systematic losses to follow-up may lead to erroneous conclusions about prevalence rates [52]**.** The use of SMS data collection methods in previous studies has resulted in low levels of drop out rates, but most of these studies have been conducted in adult populations. Our study solely examines adolescents, and it might be the case that age influences losses to follow-up. To address this issue, we will closely monitor responses to SMS messages at the commencement of the study, and determine if additional reminders are required to improve response rates.

Our proposed study will substantially improve the current understanding of the burden associated with low back pain in adolescent idiopathic scoliosis. It was previously assumed that adolescent idiopathic scoliosis was a relatively benign condition. However, recent studies have suggested that low back pain is commonly experienced by adolescents with idiopathic scoliosis. Our proposed program of research will be the first study to determine the proportion of adolescents with idiopathic scoliosis who experience acute, recurrent, and chronic low back pain, and establish the level of burden associated with these sub-groups of low back pain. It would also be beneficial to establish whether low back pain burden differs between adolescents with scoliosis and adolescents without scoliosis. Information about the level of low back pain burden in the general adolescent population is available, but such data is subject to substantial limitations [53]. *Hence, further studies are warranted to robustly establish low back pain burden in the general adolescent population.*
